# Validation and Suitability Assessment of Multiplex Mesoscale Discovery Immunogenicity Assay for Establishing Serological Signatures Using Vaccinated, Non-Vaccinated and Breakthrough SARS-CoV-2 Infected Cases

**DOI:** 10.3390/vaccines12040433

**Published:** 2024-04-18

**Authors:** Sushant Shengule, Shweta Alai, Sachin Bhandare, Sumant Patil, Manish Gautam, Bhushan Mangaonkar, Sumit Gupta, Umesh Shaligram, Sunil Gairola

**Affiliations:** Clinical Bioanalytical Department, Serum Institute of India Pvt. Ltd., Pune 411028, India; sushant.shengule@seruminstitute.com (S.S.); shweta.alai@seruminstitute.com (S.A.); m.gautam@seruminstitute.com (M.G.); umesh.shaligram@seruminstitute.com (U.S.)

**Keywords:** coronavirus, variants of concerns, herd immunity, vaccine efficacy, electrochemiluminescence

## Abstract

Antibody responses to severe acute respiratory syndrome coronavirus 2 (SARS-CoV-2) are multi-targeted and variable over time. Multiplex quantitative serological assays are needed to provide accurate and robust seropositivity data for the establishment of serological signatures during vaccination and or infection. We describe here the validation and evaluation of an electro-chemiluminescence (ECL)-based Mesoscale Discovery assay (MSD) for estimation of total and functional IgG relative to SARS-CoV-2 spike, nucleocapsid and receptor binding (RBD) proteins in human serum samples to establish serological signatures of SARS-CoV-2 natural infection and breakthrough cases. The 9-PLEX assay was validated as per ICH, EMA, and US FDA guidelines using a panel of sera samples, including the NIBSC/WHO reference panel (20/268). The assay demonstrated high specificity and selectivity in inhibition assays, wherein the homologous inhibition was more than 85% and heterologous inhibition was below 10%. The assay also met predetermined acceptance criteria for precision (CV < 20%), accuracy (70–130%) and dilutional linearity. The method’s applicability to serological signatures was demonstrated using sera samples (*n* = 45) representing vaccinated, infected and breakthrough cases. The method was able to establish distinct serological signatures and thus provide a potential tool for seroprevalence of SARS-CoV-2 during vaccination or infection.

## 1. Introduction

Amid the incidence of coronavirus disease 2019 (COVID-19), which is caused by the severe acute respiratory coronavirus 2 (SARS-CoV-2), vaccines against the variants of concern are currently being developed and licensed as boosters [1,2]. Recently, COVID-19 vaccines against XBB 1.5 variants were licensed as booster vaccines to effectively target the circulating variants of COVID-19 [3,4]. Additionally, new variants like JN.1, belonging to the parent lineage of BA.2.86 (Pirola) and EG.5 (Eris), have been recently reported globally [5,6]. The global research map for COVID-19 stresses the need for continual global sero-surveillance to measure the levels of infection and vaccine effectiveness [7,8]. Thus, the monitoring of serological responses to SARS-CoV-2 variants will be a key to developing rational vaccination strategies to combat the disease [9]. The immunodominant proteins include structural proteins, such as trimeric spike protein (S1, S2, RBD regions) and nucleocapsid (N) protein [10]. Antibody responses (IgG, IgM, and IgA) directed against the S, S1-RBD proteins confer the protective immune signatures of COVID-19, which are the key proteins in the virus entry and assembly mechanism. Antibody response to nucleocapsid antigen during infection is shown to correlate with seropositivity [9]. Since the emergence of the pandemic, 85 different serological tests have achieved authorization from the FDA [11]. However, among these 85 assays, the majority of them are monoplex assays, and very few are quantitative assays. Multiplex Serology quantitative assays, as opposed to monoplex antibody assays, are best suited for establishing the serological signatures, because they allow (a) simultaneous estimation of serological response to multiple virus protein (antigen)-specific antibodies and (b) high throughput, as well as (c) easy calibration with the international reference standards [12].

Mesoscale Discovery (MSD)’s MULTI-ARRAY^®^ electrochemiluminescence detection technology provides a quantitative multiplex immunoassay platform for such applications. The V-PLEX product line of MSD provides MULTI-SPOT^®^ (*n* = 10), which feature independent, electrically conductive, well-defined regions on coated plates with specific capture antigens/antibodies [13]. The MSD platform also offers opportunities for the development of a surrogate multiplex neutralization assay, which could simultaneously measure the ACE-2 blocking antibodies associated with multiple variants. Thus, a combination of the MSD serology assay and several surrogate neutralization assays will be the best tool to assess serological signatures [14,15].

We report here the method validation and applicability of a nine-plex MSD assay (serology and surrogate neutralization assay covering three SARS-CoV-2 antigens, namely, spike (S), receptor binding domain (RBD) of S1, and nucleocapsid (N), and the four different associated variants of spike protein and RBD-S1 protein (Wuhan, B.1.351, P.1 and B.1.1.7)), in order to study the serological signatures following infection and vaccination, and in breakthrough cases. The study involves samples collected during 15 March 2021–20 May 2022, following infection and/or vaccination. The evaluation also covered studies relevant to the WHO/NIBSC reference panel (NIBSC 20/268) for anti-SARS-CoV-2 immunoglobulins [16]. The study demonstrates the usefulness of multiplex assays in the generation of robust data on seropositivity, which will be useful during vaccination and sero-surveillance studies.

## 2. Method

### 2.1. Study Samples 

Serum samples (*n* = 45) were collected under informed consent from volunteers aged >18 years old, as reported at SIIPL, India. Details of the sera sample panel used for method validation (*n* =19, out of *n* = 45) are mentioned in Table 1. The method validation sera panel also includes the WHO/NIBSC reference panel (20/268), having panel members such as (WHO/NIBSC 20/150, 20/148, 20/140), WHO/NIBSC negative reference standard 20/142, antibody-depleted human sera and sera samples representative of negative, low, medium and high antibody concentrations [16]. Hemolytic (Hb levels at 2.02 g/dL) and lipemic sera samples (cholesterol: 172 mg/dL; triglycerides (TG): 255 mg/dL) (Haemo Service Laboratories, Hyderabad, India), were also used during the selectivity study. A total of 16 samples [6 SARS-CoV-2 positive samples; 4 (high, mid, low, or negative) WHO/NIBSC reference panel (NIBSC 20/268) members; and 6 SARS-CoV-2 negative samples] were assigned for determinations of IgG concentrations in AU/mL by performance of 6 consecutive runs [16]. The study was carried out as per the guidelines approved by the Independent Research Ethics Committee, Pune, India (IEC No. IRECP/004/2021). 

### 2.2. MSD Serology Assay Procedure: Total IgG 

The V-PLEX MSD COVID-19 Panel 7 (K15437U) (MSD, Rockville, MD, USA) kit was used for serology assay to measure antibodies against nine SARS-CoV-2 antigens, as N (Wuhan, China), S1 RBD (Wuhan, China), S1 RBD (B.1.1.7), S1 RBD (B.1.351), S1 RBD (P.1), Spike (Wuhan, China), Spike (B.1.1.7), Spike (B.1.351), Spike (P.1) [17]. The V-PLEX COVID-19 serology assay was carried out as described in the manufacturer’s manual [18]. Briefly, pre-printed 10 spot 96-well plates were blocked with the reagent A solution (MSD, Rockville, MD, USA) provided in the kit (150 µL/well) and were then incubated at room temperature for 30 min, with continuous shaking at 300 rpm. The plates were washed with 1X wash buffer (3 times; 150 µL/well). Serum sample dilutions were prepared using MSD Diluent 100 (MSD, Rockville, MD, USA). The kit provides a serum-based standard, Reference Standard 1 (MSD, Rockville, MD, USA; Lot No.: A0080286) which has defined units of measurement in AU/mL, (Supplementary Table S2) which can be used to prepare a calibration curve for the assay. Reference Standard 1 was diluted 10-fold to prepare a 7-point calibration curve fitted using a 4-PL logistics. Each serum sample, zero calibrator, and blank sample were tested in duplicate. The sample, reference standard, and assay controls (1.1, 1.2, 1.3) were added and plates were incubated at room temperature with continuous shaking at 300 rpm for 120 min. Post incubation, plates were washed 3 times with 1X wash buffer, and SULFO-TAG (anti-human IgG 1X) antibody (MSD, Rockville, MD, USA) was added to the wells (50/well). The plates were sealed and incubated at room temperature with continuous shaking at 300 rpm for 60 min. Plates were washed thrice with wash buffer, and MSD GOLD Read buffer B (150 µL) (MSD, Rockville, MD, USA) was then added to each well and the plates were read on a MESO QUICKPLEX SQ 120 reader (MSD, Rockville, MD, USA).

### 2.3. MSD Assay Procedure: ACE-2 Neutralization Assay

The V-Plex COVID-19 ACE-2 Neutralization kit (K15458U) (MSD, Rockville, MD, USA) is based on the measurement of antibodies that block the binding of ACE-2 to the SARS-CoV-2 Spike and RBD antigens. In practice, 96-well plates with multiple spots are used for the assay. Briefly, the plates were blocked with blocking buffer (MSD Blocker A) (MSD, Rockville, MD, USA), following incubation and washing with MSD wash buffer. Reference Standard 1 (MSD Calibrator), and human sera samples were added [19]. The human sera samples were analyzed at 1:10, 1:25, and 1:100 dilutions prepared using dilution buffer (MSD Diluent-100). After incubation and washing with MSD wash buffer, an ACE-2 detection antibody was added (MSD SULFO-TAG^TM^ Human ACE-2 Antibody) (MSD, Rockville, MD, USA). Subsequently, MSD GOLD^TM^ read Buffer B was added, and the plates were read using an MSD plate imager, the Meso Quick Plex SQ 120. Percentage inhibition was calculated relative to the assay calibrator (maximum 100% inhibition) using the equation below.

% Inhibition = [1 − (Average Sample ECL Signal/Average ECL signal of Calibrator 8)] ×100.

### 2.4. Assay Validation

The assay was validated as described in ICH, EMA, and US FDA guidelines on bioanalytical methods [20,21,22,23]. Assay validation was performed using a panel of sera samples. Details of the panel are provided in Table 1. 

#### 2.4.1. Specificity

Assay specificity was evaluated using a panel of 5 sera samples. These samples were evaluated, using a single dilution (1:5000) for inhibition specifically against Wuhan antigens, by mixing a neat aliquot of the virus with a sera sample in a 1:1 ratio to demonstrate the homologous antigen inhibition. Unrelated proteins, such as cross-reacting material (CRM_197-recombinant protein_), having 10 µg/mL concentration were used to demonstrate heterologous specificity. Specificity was determined based on the % inhibition of IgG against the homologous Wuhan variant and heterologous CRM_197 antigen_. 

#### 2.4.2. Selectivity

The selectivity was evaluated using several different human serum matrices: (i) matrix 1—sera from healthy volunteers (samples 10–15), (ii) matrix 2—hemolytic and lipemic matrix (samples 16–17), (iii) matrix 3—NIBSC negative sample (sample 18), and (iv) antibody-depleted human sera (sample 19), as mentioned in Table 1. 

Matrices were spiked with different concentrations of reference standard and tested at 1:1000, 1:5000 and 1:20,000 dilutions. The % recovery at each level was calculated as follows:

% Recovery = [(Observed Spike Samples − Concentration of unspiked samples)/ Concentration of Spike] × 100

An acceptance criterion of 70–130% was used for the assessment.

#### 2.4.3. Precision

The assay precision was evaluated over 3 days by different analysts using a panel of 9 different serum samples. Intra- and inter-assay precision values were reported in terms of the % coefficient of variation (% CV).

#### 2.4.4. Accuracy

Accuracy (% recovery with respect to assigned values) was assessed using a panel of sera samples (*n* = 9). Sera were tested in six different assays, using two analysts, on 3 different days. An acceptance criterion of 70–130% recovery was used for the assessment. 

#### 2.4.5. Dilutional Linearity

Dilution linearity was evaluated in twelve different runs using a panel of 9 samples. Assay was assessed using four dilutions (1:500,1:5000, 1:25,000 and 1:50,000). Recovery was calculated as the percentage difference between the observed and assigned concentrations. Dilutional Linearity was considered passing if the % CV of duplicates was <20% and recoveries were within 70–130% of the assigned values.

#### 2.4.6. Robustness

Robustness covered the evaluation of assay parameters, including sample incubation time and secondary antibody incubation time, within (±)30 min. The robustness was evaluated using nine different sera samples. The % agreement of observed versus expected concentration was determined to evaluate the impact of change in the said parameters. 

#### 2.4.7. Assay Range

The assay range was evaluated in six runs by 4-fold serial dilutions of the reference standard (Supplementary Table S2). Reference standard performance was monitored using a curve-fitting with an acceptance criterion of 70–130%. Lower and upper concentration limits showing acceptable accuracy (70–130%), precision (<20% CV) and dilutional accuracies of between 70 and 130% were selected. 

### 2.5. Standard Curve 

The standard curve was prepared using the kit’s provided reference standard. Modelling was performed using a 7-point and 4-fold dilution series of reference standard run on each assay plate. The standard fitting of the curve was performed using a 4-parameter logistic function. The assay range suggested by the manufacturer was further validated using estimates from accuracy, precision, and dilutional linearity experiments. 

### 2.6. Studies with the WHO/NIBSC Reference Panel (NIBSC 20/268)

WHO/NIBSC reference panel (NIBSC 20/268) was studied in the MSD assay to demonstrate the precision of the assay. The WHO reference panel consists of individual panel members, denoted as WHO/NIBSC reference standard 20/150 (high), 20/148 (mid), 20/140 (low) and 20/142 (negative human plasma) [16,24]. A series of six runs was performed to determine the performance of WHO/NIBSC reference panel (NIBSC 20/268) in the MSD assay. The Reference Standard 1 in V-PLEX Serology kits is calibrated against the WHO/NIBSC Reference panel (NIBSC 20/268), and the kit provides conversion factors for Wuhan Nucleocapsid protein, Wuhan RBD and Wuhan spike protein. The conversion factors 0.00236 (for W-N), 0.0217 (for W-S) and 0.0233 (for W-RBD) were used to report the units in BAU/mL for the said antigens.

### 2.7. MSD Serology Applicability Study: Development of Serological Signatures

**Figure 1:**
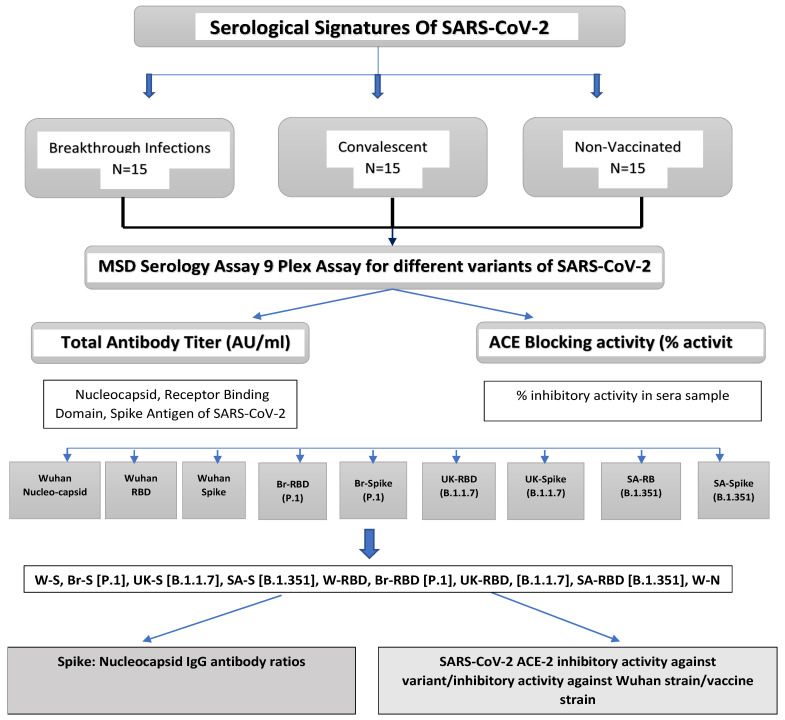
Study design followed for method applicability studies to establish serological signatures. Antigens in MSD assay is coded as follows: W-N, Wuhan Nucleocapsid; W-RBD, Wuhan receptor binding domain (RBD) protein; W-S, Wuhan Spike (S); Br-RBD [P.1], Brazil RBD; Br-S [P.1], Brazil S; UK-RBD [B.1.1.7], United Kingdom RBD; UK-S [B.1.1.7], United Kingdom S; SA-RBD [B.1.351], South Africa RBD; SA-S [B.1.351], South Africa S.

The MSD assay allows simultaneous monitoring of IgG responses (total and functional) against multiple antigens of different variants. Such profiling of antibody responses will allow the establishment of the serological signatures evident during infection and/or post-vaccination [19]. The study evaluated the suitability of the MSD assay for establishing such signatures. The study’s design schematic is provided in Figure 1. 

#### 2.7.1. Study Samples

The study uses sera samples from subjects, as reported at the Serum Institute of India Pvt. Ltd., (SIIPL), Pune, India, Occupational Health Centre (SIIPL), with the criterion of a subject’s having been over 18 years old during the period of the year 15 March 2021–20 May 2022. The sera samples were collected with written, informed consent for the collection of demographic and clinical data. A total of forty-five sera samples were selected for this study. The study’s samples represent three groups: Group 1: breakthrough infection cases; Group 2: convalescent cases and Group 3: vaccinated cases with no infection. (Figure 1). All vaccinated individuals had been immunized with the COVISHIELD^TM^ vaccine manufactured by SIIPL, Pune, India [25]. Samples were collected within 4–6 months of vaccination. The breakthrough infection and convalescent group samples were collected within 4–6 weeks and 1–2 weeks after SARS-CoV-2 infection, respectively. A confirmed case of COVID-19 was defined by having a positive result for real-time reverse transcriptase-polymerase chain reaction assay of nasal and nasopharyngeal swab specimens [26]. Blood samples were collected from the patients at the time of the first visit after the detection of symptomatic disease, and later by routine biochemical tests [27]. For total IgG estimation against nine different antigens of SARS-CoV-2, a similar MSD V-Plex COVID-19 serology assay procedure was used. 

#### 2.7.2. Ethics

The study complied with the terms of the Declaration of Helsinki, and written informed consent was obtained from all participants; the study was approved by the Independent Research Ethics Committee, Pune, India (IRECP/004/2021) [28]. 

#### 2.7.3. Data Analysis

All calculations performed during method validation were accomplished using the functionalities of Microsoft Excel. For specificity, % recovery at each level was calculated using the following formula: % recovery = [(observed spike samples − concentration of unspiked sample)/Concentration of the spike samples] × 100. 

Precision was expressed as the coefficient of variation (CV) of a series of results. Accuracy was determined by calculating the percent agreement between the established values and obtained values of each sample. Robustness was evaluated by calculating percent-agreement with the established values. In the analysis of disease severity, laboratory parameters were compared between the convalescent group and breakthrough infected group by Student’s t-test. All *p* values of <0.05 were considered significant. 

Serological signatures were established for total and functional IgG using the following parameters:

(a) For total IgG: Ratios were determined as IgG concentration against SARS-CoV-2 protein (S-1RBD, spike)/IgG concentration against nucleocapsid protein. Such ratios were determined for each variant.
(b) For functional IgG: Ratios were determined for ACE inhibition activity observed for circulating variant/inhibition activity observed for the Wuhan variant.

The ratios (a and b), IgG concentrations and % ACE blocking activity for all of the three groups, vaccinated, convalescent, and breakthrough cases, were compared using one-way ANOVA (Kruskal–Wallis test). All *p* values of <0.05 were considered significant. 

## 3. Results

### 3.1. MSD Assay Validation and Characterization

Sera Panel Establishment

Target values for the sera panel were established using data from six different runs. The established values for the sera panel are provided in Supplementary Table S3.

### 3.2. Reference Standard Curve

**Figure 2:**
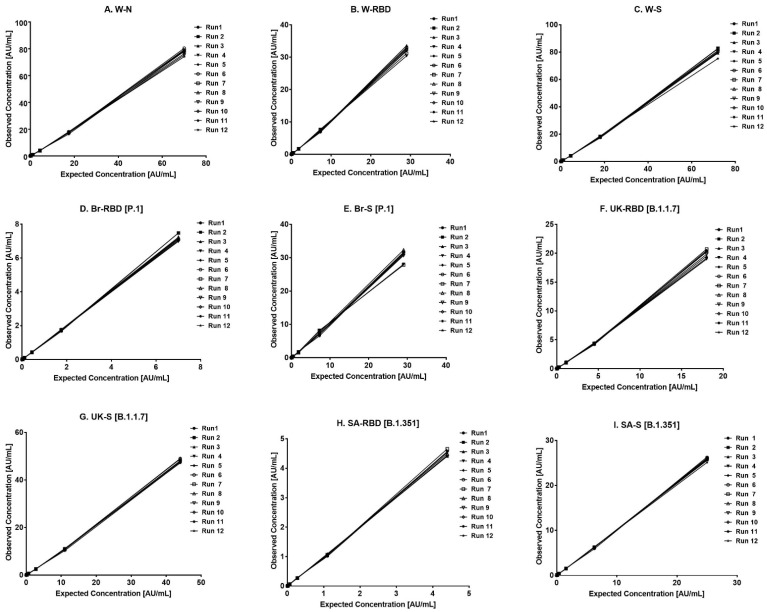
Standard curve range of the assay. (**A**–**I**) represents the assay range for the nine antigens. The X-axis represents the expected concentration (AU/mL) whereas the Y-axis represents the obtained concentration (AU/mL). Data are representative of 12 runs. Abbreviations: W-N, Wuhan Nucleocapsid; W-RBD, Wuhan receptor binding domain (RBD); W-S, Wuhan Spike (S); Br-RBD [P.1], Brazil RBD; Br-S [P.1], Brazil S; UK-RBD [B.1.1.7], United Kingdom RBD; UK-S [B.1.1.7], United Kingdom S; SA-RBD [B.1.351], South Africa RBD; SA-S [B.1.351], South Africa S.

Figure 2 shows the verified reference standard range for the assay. Curve back-fitted recoveries were used to establish the linearity, wherein values in the range of 70–130% were observed for all the calibration levels. The estimates from accuracy, precision, and robustness experiments were also considered to support the upper and lower limits of the assay range. Table 2 provides the UL and LL of quantifications, as observed in different validation parameters. 

### 3.3. Specificity

Specificity was demonstrated in Table 3 for all of the nine antigens in the panel using inhibition experiments. The addition of homologous antigens resulted in >75% inhibition. The heterologous inhibition found <25% inhibition, indicating the high specificity of the assay.

#### 3.3.1. Precision

The precision experiments were carried out using a panel of serum samples representing different concentration levels. Assay precision values (different days, different analysts) were determined to be below 20% (Table 4). Precision data were also used to support the assay range, wherein acceptable precision was observed in the range of 0.01914 to 86.1 AU/mL for W-N, 0.00777 to 35.4 AU/mL for W-RBD, 0.01960 to 82.7 AU/mL for W-S, 0.00119 to 7.9 AU/mL for Br-RBD (P.1), 0.00700 to 32.3 AU/mL for Br-S (P.1), 0.00337 to 23.0 AU/mL for UK-RBD (B.1.1.7), 0.01071 to 49.0 AU/mL for UK-S (B.1.1.7), 0.00077 to 4.8 AU/mL for SA-RBD (B.1.351), and 0.00682 to 26.3 AU/mL for SA-S (B.1.351) (Table 2 and Table 4).

#### 3.3.2. Accuracy

Assay accuracy values for all nine antigens, using different serum samples, were determined to be in the range of 70–130% (Table 4). 

Based on the sera panel, accuracy-based LLs and ULs ranged from 0.01914 to 86.1 AU/mL for W-N, 0.00777 to 35.4 AU/mL for W-RBD, 0.01960 to 82.7 AU/mL for W-S, 0.00119 to 7.9 AU/mL for Br-RBD (P.1), 0.00700 to 32.3 AU/mL for Br-S (P.1), 0.00337 to 23.0 AU/mL for UK-RBD (B.1.1.7), 0.01071 to 49.0 AU/mL for UK-S (B.1.1.7), 0.00077 to 4.8 AU/mL for SA-RBD (B.1.351), and 0.00682 to 26.3 AU/mL for SA-S (B.1.351) (Table 2).

#### 3.3.3. Selectivity

The selectivity of the method was determined using different serum matrices, including SARS-CoV-2 negative samples, NIBSC negative samples, hemolytic sera, antibody-depleted human sera, and lipemic sera. The assay showed acceptable recoveries (70–130%) in all the matrices for all the antigens (Table 5). No interference was observed in the assay for hemolytic and lipemic matrices, covering up to 2.02 g/dL of hemoglobin and 275 mg/mL of total cholesterol, respectively. 

#### 3.3.4. Robustness

The robustness of the assay was studied using a panel of sera samples. Critical steps of the assay including Ag-Ab incubation time and sulfo-tag (secondary antibody) incubation time were studied for robustness. The results demonstrated that the critical steps of the assay are robust; the IgG concentrations were found to be within the acceptance criteria, with deliberate variations in the said parameters (Table 6). 

### 3.4. Dilution Linearity

**Figure 3:**
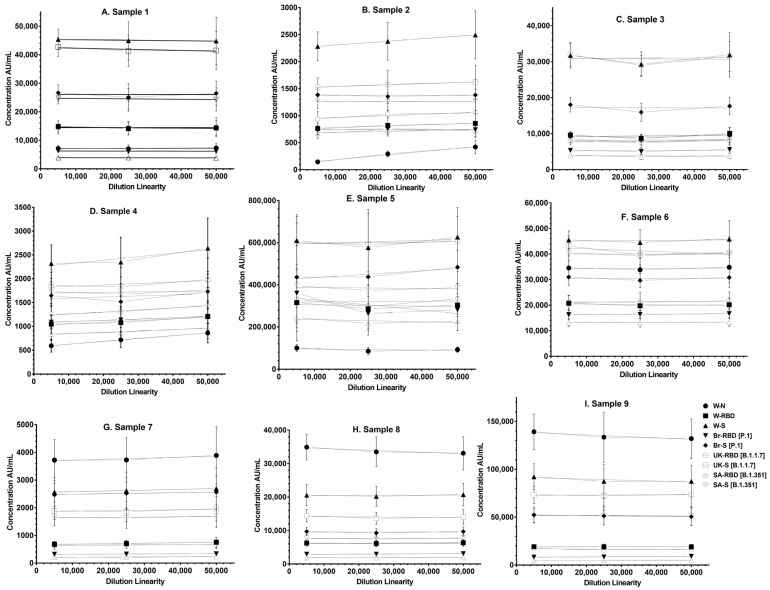
Dilution linearity of the assay. (**A**–**F**) represent dilution linearity graphs observed for infected sera samples. (**G**–**I**) represent dilution linearity data for NIBSC samples. The X-axis represents the sample’s dilutions, and the Y-axis represents the concentration observed in (AU/mL). The dotted line in the figure represents the 95% confidence interval. Abbreviations: W-N, Wuhan Nucleocapsid; W-RBD, Wuhan receptor binding domain (RBD); W-S, Wuhan Spike (S); Br-RBD [P.1], Brazil RBD; Br-S [P.1], Brazil S; UK-RBD [B.1.1.7], United Kingdom RBD; UK-S [B.1.1.7], United Kingdom S; SA-RBD [B.1.351], South Africa RBD; SA-S [B.1.351], South Africa S.

The panel samples were tested in twelve independent runs across a series of sera samples ranging from a dilution of 1:500 to a dilution of 1:50,000. No loss in dilution integrity was observed with 1:5000, 1:25,000 and 1: 50,000 dilution ranges, as recorded for all antigens (Figure 3).

### 3.5. Assay Range

The assay range was selected based on the estimates of precision, and accuracy. The LL and UL of the assay range were established as bounding ranges from 0.01914 to 86.1 AU/mL for W-N, 0.00777 to 35.4 AU/mL for W-RBD, 0.01960 to 82.7 AU/mL for W-S, 0.00119 to 7.9 AU/mL for Br-RBD (P.1), 0.00700 to 32.3 AU/mL for Br-S (P.1), 0.00337 to 23.0 AU/mL for UK-RBD (B.1.1.7), 0.01071 to 49.0 AU/mL for UK-S (B.1.1.7), 0.00077 to 4.8 AU/mL for SA-RBD (B.1.351), and 0.00682 to 26.3 AU/mL for SA-S (B.1.351) (Table 2).

### 3.6. Studies with the WHO/NIBSC Reference Panel (NIBSC 20/268)

The WHO/NIBSC reference Panel (NIBSC 20/268) is recommended by the WHO for assessment and development of assays used in the detection and quantitation of antiSARS-CoV-2 antibodies [16]. The panel provides the calibrations for spike, RBD and Nucleocapsid antibodies for Wuhan antigens only. WHO/NIBSC reference panel (NIBSC 20/268) performance was assessed in the assay in multiple runs over different days and analysts. The study reports values in AU/mL (calculated against MSD reference standard 1) and BAU/mL (determined against WHO/NIBSC reference standard (NIBSC 20/136) using conversion factors reported by MSD for Wuhan antigens only) [18]. These factors will be useful for the harmonization of assays across different laboratories (Table 7).

#### 3.6.1. Method Applicability for Development of Serological Signatures for Nucleocapsid, RBD and Spike Protein of Ancestral Strain 

A total of forty-five sera samples were analyzed for total IgG estimation against N, S and S1 RBD antigens, using Mesoscale Discovery (MSD) COVID-19 serology assay panel 7 (Supplementary Table S3). The results showed significantly (*p* < 0.001) higher antibodies against N protein in the convalescent patient cohort [68,887 AU/mL (1088–455,149 AU/mL)], as compared to the breakthrough infected group [568 AU/mL (47–2649 AU/mL)] and vaccinated non-infected group [361 AU/mL (160–1040 AU/mL)] [29]. This is consistent with previous reports wherein antibody levels for nucleocapsid are shown to correlate with viral loads [30,31]. The trend in vaccinated and non-vaccinated samples was further found to be consistent with RT-PCR and other virologic parameters associated with the disease (Table 8). 

Antibodies against spike protein subunits S1 RBD have been reported to correlate with virus neutralization activities [32,33]. In the vaccinated group, S1 RBD antibodies were found to be in the range of [1936 AU/mL (512–5426 AU/mL)]. These levels were along expected lines, as these samples were collected 4–6 months post-vaccination [34]. Further, these levels are consistent with reports that antibodies are known to wane with time [35]. A significantly (*p* < 0.01) higher level of antibodies was observed against S1 RBD in the breakthrough infection group [35,780 AU/mL (918–459,708 AU/mL)], as compared to the vaccinated group. 

A similar trend was observed for Spike [S1 and S2] antigens, wherein a significant (*p* < 0.001) increase in IgG antibodies was observed in the vaccinated breakthrough infected patient cohort [94,780 AU/mL (4515–1,170,950 AU/mL)], as compared to the convalescent group [49,670 AU/mL (585–1,420,159 AU/mL)] [36]. Further, this increase is consistent with reports on robust recall responses in vaccinated subjects [37]. This is, further, consistent with other virologic characterization, wherein the breakthrough subjects did not experience severe infection outcomes and hospitalization was limited. 

#### 3.6.2. Serological Signatures: Immune Responses to Different Variants-Total IgG

Multiplex assays such as MSD allow simultaneous measurement of IgG responses against Wuhan and variant strains. The samples were evaluated for IgG concentrations against Brazil SA and UK variants. It was noted that all of the subjects received vaccines containing the Wuhan variant [38]. The assay predicted immune response against the different variants in the vaccinated group, wherein the levels of antibodies against the Wuhan, Brazil, SA, and UK variants of the spike antigen were 7211 AU/mL (2433–15,193 AU/mL), 3438 AU/mL (976–10,680 AU/mL), 3517 AU/mL (1577–7598 AU/mL) and 5040 AU/mL (2045–14,085 AU/mL), respectively. For RBD antigen, the level of antibodies against the Wuhan, Brazil, SA, and UK variants were 1936 AU/mL (512–5426 AU/mL), 1495 AU/mL (634–5440 AU/mL), 1117 AU/mL (287–3923 AU/mL) and 2083 AU/mL (596–6248 AU/mL) respectively [39].

The sera of breakthrough and convalescent subjects are representative of infections during the second wave of the pandemic. A significantly higher number of antibodies (median) against Brazil, SA, and UK variants was observed in the breakthrough group. The trend of the levels was UK > Brazil > SA. This trend is consistent with published reports wherein vaccination with Wuhan did protect against different variants [40]. This was further supported by reduced levels of nucleocapsid antibody levels in these groups. 

The sera of convalescent groups showed antibodies against different variants, with a trend of UK > Brazil > SA, respectively (Table 9). 

#### 3.6.3. Serological Signatures for Functional Antibodies

ACE-2 blocking antibodies against variants were measured in all three groups. It was noted that the vaccinated and non-infected group reported the lowest inhibitory activities against all the variants. This was found to be consistent with previous reports wherein neutralizing antibodies were reported to wane with time [31]. The highest inhibitory activity (*p* < 0.05, as compared to the vaccinated non-infected group) was reported in the breakthrough-infected group. The levels of inhibition were UK > SA > Brazil for the spike antigen and SA > UK > Br for the RBD antigen. This trend is consistent with published reports on robust recall responses associated with COVID-19 vaccines [31,41]. 

The convalescent group also reported significantly higher inhibitory activities (*p* < 0.05, as compared to the vaccinated and non-infected group) against RBD and spike antigen. The order of the levels of inhibition was UK > Brazil for RBD and spike antigens (Table 10). However, the inhibitory activities reported in the convalescent group were lower, as compared to the breakthrough-infected group. 

### 3.7. Method’s Applicability in Prediction of Distinct Signatures

#### Spike Protein: Nucleocapsid IgG Antibody Concentration Ratios Were Distinctive among Groups

The ratios of IgG concentration for the spike and nucleocapsid protein [S/N] and RBD and nucleocapsid protein [R/N] for the vaccinated and non-vaccinated infected groups were compared [42]. These ratios were found to be significant (*p* < 0.001) among the groups, wherein the highest ratios were observed, in ascending order, for the breakthrough > vaccinated > convalescent groups. These ratios were also monitored for RBD and Spike IgG concentrations of different variants and a similar pattern was observed (Table 9). 

### 3.8. Method’s Applicability in Developing Profiles against Variants

All of the subjects associated with vaccinated and breakthrough cases had received the vaccine manufactured using the Wuhan strain. Sera samples were profiled for total and functional IgG antibodies against different variants [12]. The ratio of antibodies (variant/antibody concentration against vaccine strain) was determined for each of the groups. These ratios were found to be significant (*p* < 0.05) among the groups, wherein the highest ratios were observed, in ascending order, for the breakthrough > convalescent > vaccinated groups. These ratios were also monitored for Spike IgG concentrations of different variants, and a similar pattern was observed for all variants except the UK variant; the highest ratios were observed, in ascending order, for the convalescent > breakthrough > vaccinated groups (Table 11).

### 3.9. Hematological Parameters in Convalescent/Breakthrough Subjects 

Hematological profiles of the convalescent (*n* = 15) and breakthrough (*n* = 15) groups were compared in order to study associations with serological signatures. The results suggest significant differences in CRP (*p* < 0.01), D-Dimer (*p* < 0.001), Ferritin (*p* < 0.001) and LDH (*p* < 0.001) levels for the groups of convalescent and breakthrough cases along the expected lines (Table 12). It was noted that the values for the breakthrough group were significantly lower for all of these parameters, suggesting reduced severity of the disease in vaccinated subjects [43,44,45,46]. This is further consistent with the trends observed in serology, wherein breakthrough groups reported significantly reduced levels of nucleocapsid antibodies, as compared to the convalescent group [47].

## 4. Discussion

A COVID-19 vaccine based on the spike protein continues to protect against negative disease outcomes, even in the presence of emerging variants [48]. Serological responses to SARS-CoV-2 infection are multi-targeted and therefore bring challenges in effective monitoring. Multiplex assays bring opportunities to simultaneously estimate antibody responses against multiple proteins comprising ancestral as well as variant strains. Such multi-dimensional data further offer opportunities for the establishment of serological signatures. We report here the validation of a multiplex MSD platform-based serological assay and its applicability in evaluation of serological signatures of SARS-CoV-2. The signatures were identified for total and functional IgG levels against parents and different variants. The functional IgG was determined using ACE receptor-blocking antibodies on the MSD platform. A neutralization assay is reported for the quantification of virus-neutralizing antibodies [49]. 

The performance of any multiplex assay depends on many factors [12]. The MSD assay was therefore validated with parameters from the guidelines of the US FDA, EMA, and ICH guidance on bioanalytical methods. This study reports for the very first time the performance evaluation of an MSD assay for different variants. The assay’s performance against different variants was slightly variable. It was noted that the assay showed relatively high variability against Brazil and SA variants [50]. This could be due to multiple reasons, as the technology involves spotting and single-detection antibodies for all of the variants. It is recommended that each laboratory should perform validation and establish the performance metrics in their laboratories using international reference standards, as this will allow the pooling of data across the laboratories. This study reports the use of WHO/NIBSC reference panel (20/268) in the multiplex method, which could be used to perform inter-lab comparison studies, studying the lab-to-lab variabilities.

Serological signatures involving multiple proteins of different variants offer a comprehensive understanding of serologic responses against infection, vaccination and recall responses. For applicability to sero-signatures, results were reported from a pilot scale study using 45 seropositive individuals, representing vaccination, infection and vaccinated individuals following breakthrough infection. The vaccinated samples reflect immunization with the COVISHIELD™ vaccine. The vaccine is based on an adeno-vector platform (ChAdOx1 ncov-19 coronavirus particles) which is a recombinant, replication-deficient chimpanzee adenovirus vector encoding the SARS-CoV-2 spike glycoprotein of the Wuhan variant [38]. The study uses sera samples collected during 2021–2022, and thus representing infections with the Delta or Omicron variants of concern (VOCs). The method was able to establish distinct serological signatures associated with infection, vaccination, and breakthrough responses. Antibodies against nucleocapsid proteins were found to be highly indicative of infection in cases in which the breakthrough and infection groups showed significantly higher antibodies against the nucleocapsid protein. The diagnostic significance of antibodies against nucleocapsid protein has already been reported and established [10]. An attempt was also made to study the ratios of antibodies against nucleocapsid and spike protein in these groups. It was noted that the ratios were also predictive of distinct signatures in the groups used in the study. Antibodies against S protein and the RBD of SARS-CoV-2 collectively serve as a target for the development of vaccines and therapy [51,52]. The assay was able to predict different levels of reactivities to spike protein and S1-RBD in different groups. Vaccinated breakthrough cases showed a significantly higher number of antibodies against spike protein and RBD, supporting the finding of a strong recall response with the vaccine. Further, this is consistent with reports on COVID-19 vaccines wherein a robust recall response was reported in breakthrough cases [53]. 

Several variants of concern (VOC) of SARS-CoV-2 have emerged [54]. There is an urgent need to quantify the breadth of immune responses generated by any vaccine against these variants [35,55]. Between the three groups, vaccinated breakthrough infection showed the highest median antibody titer values of IgG, followed by convalescent and vaccinated non-infected, for all variants. This further supports the reports of robust recall responses associated with COVID-19 vaccines. The assay reported antibodies against variants in the order of Wuhan > Br >UK > SA for both spike and RBD, thereby supporting the reports of variable degrees of protection against variants of SARS-CoV-2. 

Neutralizing or functional antibodies against the SARS-CoV-2 virus were reported using different assay platforms, including plaque reduction neutralization test (PRNT), microneutralization test and pseudo neutralization test [56]. However, all of these tests are laborious and require a considerable amount of serum. Determination of functional IgG levels using surrogate neutralization tests such as the MSD ACE receptor inhibition assay offers opportunities for high throughput and simultaneous estimation against different variants, and, most importantly, uses a minimal amount of serum sample. The study analyzed ACE2 binding inhibition within serum samples from vaccinated infected individuals. The percentage-inhibition of ACE-2 receptor binding to all four variants for the vaccinated breakthrough-infected cohort was significantly higher than for the convalescent group. ACE2 binding inhibition activities in sera against the Wuhan strain were found to be highest, as compared to other variants. This is along expected lines, as variants have reported specific mutations which enhance RBD-ACE2 receptor affinity [57]. Additionally, ACE inhibitory activities also showed a positive correlation with the level of IgG concentrations against the spike protein. Therefore, the signatures reported by assay were able to distinctively classify the groups of convalescent, breakthrough, and vaccinated groups. 

The present study has some limitations concerning the number of samples in this study. The study reports from 45 subjects, which is a very limited number. Nevertheless, the study provides sufficient evidence of the capability of the multiplex methods to establish serological signatures. Another limitation of the study is that the study’s analyses were performed on a panel of sera samples available at occupational health centres. Though suitable care was taken to identify the best panel of samples for this study, a time course study would have further helped to establish the kinetics of the immune responses. The study uses panel 7 MSD kits, which were the most up to date kits available during the tenure of this study. It should be noted that recently, MSD has also introduced kits with the most current XBB variant in the panel. It will be interesting to profile the sera samples further against the XBB variant [8,40]. It is also of note that, at the time of data analysis, WHO/NIBSC also introduced a new reference standard for VOCs, WHO/NIBSC 22/270. It will be helpful to develop further calibration factors against the international standard for SARS-CoV-2 to allow reporting of the results in international units. 

Multiplex quantitative assays such as MSD can play an important role in sero-surveillance studies by providing robust data on antibodies against multiple proteins of SARS-CoV-2 variants. Further, such data will be helpful in the monitoring of infection and the impacts of vaccines on circulating variants. 

## 5. Conclusions

The MSD multiplex assay was validated according to ICH, EMA and US FDA recommendations for bioanalytical methods. The assay demonstrated high specificity, selectivity, precision, and accuracy in quantifying IgG concentrations against multiple proteins of SARS-CoV-2 variants in human serum matrices. The range and sensitivity of the assay further supported its use in regions of low seroprevalence. The study also reports results from a pilot scale study wherein the method was able to establish distinct serological signatures among different groups of vaccinated, infected, and breakthrough cases. The study overall supports the use of quantitative multiplex assays such as MSD in establishing serological profiles during vaccination and/or infection.

## Figures and Tables

**Table 1:** Sera sample panel used for assay validation.

Sr. No.	Sample ID	Sample Description	Test Details
1	Sample 1	SARS-CoV-2 positive samples	Samples used for specificity, accuracy, precision, robustness, and stability study
2	Sample 2		
3	Sample 3		
4	Sample 4		
5	Sample 5		
6	Sample 6		
7	Sample 7	WHO/NIBSC reference panel (NIBSC 20/268)	
8	Sample 8		
9	Sample 9		
10	Sample 10	SARS-CoV-2 negative samples	Samples used for selectivity study
11	Sample 11		
12	Sample 12		
13	Sample 13		
14	Sample 14		
15	Sample 15		
16	Sample 16	Hemolytic and lipemic samples	
17	Sample 17		
18	Sample 18	WHO/NIBSC negative reference standard (20/142)	
19	Sample 19	Sigma ADHS	

**Table 2:** Assay range. Lower and upper limits of quantification observed from different validation parameters supporting the assay range.

Antigen	Calibration Curve Range (AU/mL)		Precision (AU/mL)		Accuracy (AU/mL)		Robustness (AU/mL)		Selectivity (AU/mL)	
	Lower Limit	Upper Limit	Lower Limit	Upper Limit	Lower Limit	Upper Limit	Lower Limit	Upper Limit	Lower Limit	Upper Limit
W-N	0.01710	70	0.01914	86.1	0.01914	86.1	0.01957	80.8	0.02022	83.2
W-RBD	0.00708	29	0.00777	35.4	0.00777	35.4	0.00812	33.1	0.00813	35.6
W-S	0.01760	72	0.01960	82.7	0.01960	82.7	0.02110	82.1	0.02212	86.2
Br-RBD (P.1)	0.00171	7	0.00119	7.9	0.00119	7.9	0.00152	7.4	0.00210	7.6
Br-Spike (P.1)	0.00708	29	0.00700	32.3	0.00700	32.3	0.00417	31.0	0.00732	32.7
UK-RBD (B.1.1.7)	0.00439	18	0.00337	23.0	0.00337	23.0	0.00044	21.3	0.00453	21.8
UK-S (B.1.1.7)	0.01070	44	0.01071	49.0	0.01071	49.0	0.01300	50.2	0.01342	52.7
SA-RBD (B.1.351)	0.00107	4.4	0.00077	4.8	0.00077	4.8	0.00086	5.4	0.00108	4.6
SA-S (B.1.351)	0.00610	25	0.00682	26.3	0.00682	26.3	0.00629	26.3	0.00733	27.7

Abbreviations: W-N, Wuhan Nucleocapsid; W-RBD, Wuhan receptor binding domain (RBD); W-S, Wuhan Spike (S); Br-RBD [P.1], Brazil RBD; Br-S [P.1], Brazil S; UK-RBD [B.1.1.7], United Kingdom RBD; UK-S [B.1.1.7], United Kingdom S; SA-RBD [B.1.351], South Africa RBD; SA-S [B.1.351], South Africa S.

**Table 3:** Specificity. Homologous (Ho) and heterologous (He) inhibition were determined for nine antigens. Reported values are averages from three different runs. All % CV observed for these percentages were below 10%.

Sample	% Inhibition																	
	W-N		W-RBD		W-S		Br-RBD [P.1]		Br-S [P.1]		UK-RBD [B.1.1.7]		UK-S [B.1.1.7]		SA-RBD [B.1.351]		SA-S [B.1.351]	
	Ho	He	Ho	He	Ho	He	Ho	He	Ho	He	Ho	He	Ho	He	Ho	He	Ho	He
Sample 1	89	1	88	2	89	1.3	89	2	89	4	88	1.6	88	2.3	88	1.3	89	2
Sample 3	89	0.6	88	3.3	88	2	89	1.6	89	3.3	88	0.3	87	1	87	4	88	1
Sample 6	89	0.6	88	1.3	89	0.6	89	2.6	89	2	88	1	89	0.6	88	1.3	88	0.3
Sample 8	90	2	89	1	89	0	89	0	90	2.3	89	1.3	89	1.6	88	0.3	89	3.6
Sample 9	90	1	90	1.3	90	1.3	89	0	90	3.6	89	2.3	89	2.3	88	0	89	0.6

Abbreviations: W-N, Wuhan Nucleocapsid; W-RBD, Wuhan receptor binding domain (RBD); W-S, Wuhan Spike (S); Br-RBD [P.1], Brazil RBD; Br-S [P.1], Brazil S; UK-RBD [B.1.1.7], United Kingdom RBD; UK-S [B.1.1.7], United Kingdom S; SA-RBD [B.1.351], South Africa RBD; SA-S [B.1.351], South Africa S.

**Table 4:** Precision and accuracy estimates. Analyst- and day-wise precision and accuracy estimates. Precision is reported in terms of mean concentration values. Values in parenthesis represent % CV observed for mean concentration in different runs. Accuracy is represented in terms of percent recovery. Values in parenthesis represent % CV observed for mean recovery in different runs.

	Precision																	
	Analyst Mean Concentration (% CV)									Days Mean Concentration (% CV)								
	W-N	W-RBD	W-S	Br-RBD [P.1]	Br-S [P.1]	UK-RBD [B.1.1.7]	UK-S [B.1.1.7]	SA-RBD [B.1.351]	SA-S [B.1.351]	W-N	W-RBD	W-S	Br-RBD [P.1]	Br-S [P.1]	UK-RBD [B.1.1.7]	UK-S [B.1.1.7]	SA-RBD [B.1.351]	SA-S [B.1.351]
Sample 1	7396 (15)	14,433 (6)	44,740 (6)	6310 (5)	27,214 (10)	13,961 (6)	41,709 (5)	3939 (6)	24,505 (6)	6865 (15)	15,174 (12)	44,728 (10)	6540 (8)	27,259 (9)	13,689 (15)	42,756 (8)	4063 (9)	24,653 (10)
Sample 2	222 (16)	758 (10)	2294 (11)	691 (10)	1324 (9)	907 (14)	1520 (12)	624 (13)	1208 (12)	192 (16)	749 (14)	2238 (13)	722 (12)	1332 (9)	928 (17)	1486 (13)	656 (14)	1218 (12)
Sample 3	9063 (11)	8983 (6)	28,870 (8)	5052 (7)	16,563 (19)	7215 (7)	29,474 (6)	3525 (9)	7687 (8)	8967 (11)	9982 (11)	30,785 (13)	5671 (11)	17,900 (18)	8026 (14)	31,378 (10)	4025 (14)	8150 (11)
Sample 4	692 (18)	947 (12)	2335 (12)	1066 (11)	1571 (11)	1316 (12)	1843 (12)	789 (12)	1632 (13)	681 (18)	1147 (19)	2278 (16)	1099 (15)	1610 (12)	1112 (19)	1807 (15)	832 (16)	1678 (16)
Sample 5	81,051 (13)	210,240 (19)	442,068 (15)	224,813 (12)	323,709 (15)	224,079 (14)	424,005 (15)	180,490 (15)	266,745 (16)	87,937 (13)	229,179 (17)	530,518 (16)	258,502 (19)	358,398 (15)	220,553 (18)	431,782 (20)	195,319 (15)	284,238 (16)
Sample 6	35,069 (6)	19,350 (12)	43,360 (10)	16,267 (6)	30,816 (5)	19,890 (16)	41,374 (9)	13,116 (9)	37,770 (12)	34,724 (8)	20,514 (11)	44,561 (10)	18,184 (11)	31,475 (18)	21,403 (14)	43,004 (10)	14,074 (11)	41,798 (13)
Sample 7	3345 (20)	625 (15)	2446 (16)	301 (13)	2227 (18)	587 (16)	1828 (17)	197 (15)	1535 (15)	3512 (20)	756 (19)	2420 (20)	289 (17)	2174 (17)	536 (19)	1941 (18)	191 (19)	1554 (20)
Sample 8	34,699 (12)	6283 (13)	19,729 (17)	2738 (13)	9254 (18)	5838 (11)	13,624 (16)	1776 (12)	7260 (16)	37,157 (12)	6707 (12)	20,923 (14)	2997 (15)	9605 (14)	6188 (13)	14,384 (13)	1889 (12)	7685 (14)
Sample 9	109,976 (15)	19,447 (14)	73,605 (15)	8693 (16)	45,103 (17)	18,467 (15)	69,539 (15)	4974 (15)	48,400 (16)	122,645 (15)	21,138 (14)	83,563 (17)	9276 (14)	55,206 (16)	19,668 (15)	78,635 (16)	5314 (13)	56,901 (13)
	**Accuracy**																	
	**Analyst-%-Recovery (% CV)**									**Days-%-Recovery (% CV)**								
Sample 1	94 (15)	101 (6)	104 (6)	101 (5)	110 (10)	101 (6)	104 (5)	104 (6)	104 (6)	94 (15)	100 (12)	100 (10)	101 (8)	106 (9)	100 (15)	102 (8)	103 (9)	100 (10)
Sample 2	105 (16)	100 (10)	95 (11)	105 (10)	93 (9)	112 (14)	97 (12)	116 (13)	93 (12)	105 (16)	93 (14)	88 (13)	102 (12)	89 (9)	105 (17)	90 (13)	110 (14)	88 (12)
Sample 3	87 (11)	93 (6)	95 (8)	77 (7)	107 (19)	94 (7)	95 (6)	96 (9)	89 (8)	87 (11)	93 (11)	91 (13)	79 (11)	99 (18)	93 (14)	94 (10)	95 (14)	85 (11)
Sample 4	75 (18)	97 (12)	90 (12)	104 (11)	87 (11)	110 (12)	92 (12)	115 (12)	89 (13)	75 (18)	91 (19)	84 (16)	100 (15)	84 (12)	103 (19)	86 (15)	109 (16)	84 (16)
Sample 5	93 (13)	89 (19)	91 (15)	82 (12)	85 (15)	88 (14)	90 (15)	102 (15)	82 (16)	93 (13)	92 (17)	102 (16)	90 (19)	91 (15)	90 (18)	94 (20)	107 (15)	81 (16)
Sample 6	88 (8)	97 (8)	92 (10)	95 (10)	89 (4)	100 (9)	94 (10)	94 (9)	88 (12)	88 (8)	96 (11)	88 (10)	99 (11)	98 (18)	99 (14)	91 (10)	95 (11)	86 (13)
Sample 7	73 (20)	95 (15)	94 (16)	103 (13)	93 (18)	104 (16)	97 (17)	106 (15)	101 (15)	73 (20)	90 (19)	82 (20)	90 (17)	82 (17)	88 (19)	91 (18)	94 (19)	92 (20)
Sample 8	84 (12)	87 (13)	85 (17)	90 (13)	91 (18)	114 (11)	87 (16)	90 (12)	88 (16)	84 (12)	86 (12)	81 (14)	89 (15)	87 (14)	112 (13)	83 (13)	89 (12)	84 (14)
Sample 9	91 (15)	87 (14)	86 (15)	89 (16)	80 (17)	91 (15)	91 (15)	90 (15)	86 (16)	91 (15)	92 (14)	88 (17)	93 (14)	86 (16)	94 (15)	95 (16)	96(13)	90 (16)

Abbreviations: W-N, Wuhan Nucleocapsid; W-RBD, Wuhan receptor binding domain (RBD); W-S, Wuhan Spike (S); Br-RBD [P.1], Brazil RBD; Br-S [P.1], Brazil S; UK-RBD [B.1.1.7], United Kingdom RBD; UK-S [B.1.1.7], United Kingdom S; SA-RBD [B.1.351], South Africa RBD; SA-S [B.1.351], South Africa S.

**Table 5:** Method Selectivity. Percent recoveries in different serum matrices. Reference standard was spiked in different matrices and percentage values for recovery were calculated. Values represent mean recoveries from six independent runs. Percent variability among runs was below 20%.

Samples	Sample Description	Reference Standard Spike Level	% Recovery								
			W-N	W-RBD	W-S	Br-RBD [P.1]	Br-S [P.1]	UK-RBD [B.1.1.7]	UK-S [B.1.1.7]	SA-RBD [B.1.351]	SA-S [B.1.351]
Sample 10	SARS-CoV-2 negative samples	High	90	88	87	87	92	98	85	98	95
		Mid	103	107	105	92	109	130	106	120	118
		Low	99	108	104	125	102	123	123	112	128
Sample 11		High	91	86	89	87	94	97	89	94	99
		Mid	108	103	109	115	111	88	105	123	115
		Low	120	110	82	105	75	95	80	107	74
Sample 12		High	92	89	94	89	91	113	105	96	104
		Mid	114	113	116	127	97	125	86	81	83
		Low	105	95	86	102	86	122	118	86	89
Sample 13		High	90	93	89	96	95	96	89	96	96
		Mid	86	91	98	85	95	75	101	90	102
		Low	95	97	81	82	84	80	73	83	104
Sample 14		High	91	91	88	90	93	99	88	103	96
		Mid	102	98	97	111	106	104	103	130	114
		Low	124	95	84	106	95	123	124	112	115
Sample 15		High	87	90	87	104	92	95	88	102	96
		Mid	105	128	105	122	124	120	115	125	114
		Low	97	109	121	108	118	73	85	123	115
Sample 16	Hemolytic and lipemic samples	High	116	83	103	127	97	86	80	70	90
		Mid	105	86	108	115	90	82	76	75	96
		Low	113	95	116	128	96	81	71	80	88
Sample 17		High	86	85	88	101	97	94	89	118	95
		Mid	86	130	110	111	116	118	119	111	123
		Low	86	70	111	125	75	78	118	120	73
Sample 18	WHO/NIBSC negative (20/142) Sample	High	92	82	94	97	95	95	92	87	95
		Mid	96	104	99	108	103	90	106	104	106
		Low	82	71	104	112	101	82	89	125	90
Sample 19	Sigma antibody-depleted human serum	High	88	87	92	102	92	95	89	96	93
		Mid	90	105	95	106	99	94	95	97	97
		Low	89	100	94	112	105	102	91	96	94

Abbreviations: W-N, Wuhan Nucleocapsid; W-RBD, Wuhan receptor binding domain (RBD); W-S, Wuhan Spike (S); Br-RBD [P.1], Brazil RBD; Br-S [P.1], Brazil S; UK-RBD [B.1.1.7], United Kingdom RBD; UK-S [B.1.1.7], United Kingdom S; SA-RBD [B.1.351], South Africa RBD; SA-S [B.1.351], South Africa S.

**Table 6:** Assay Robustness. Critical steps of the assay were challenged with deliberate variations, and the impacts were studied using a sera panel (*n* = 9). Range presented in the table represents the highest and lowest recovery observed in the sera panel. An acceptance criteria of 70–130% was used for assessment.

Percent Recoveries with Respect to Assigned Values				
Antigen	Ag-Ab Incubation		Sulfo-Tag Incubation	
	150 min	90 min	90 min	30 min
W-N	72–121	72–115	70–121	93–119
W-RBD	89–130	88–130	83–126	100–129
W-Spike	93–125	93–125	86–124	101–122
Br-RBD [P.1]	90–114	84–115	81–110	83–114
Br-Spike [P.1]	70–126	71–115	85–128	92–124
UK-RBD [B.1.1.7]	88–113	83–130	80–129	87–126
UK-S [B.1.1.7]	97–112	90–121	88–122	98–123
SA-RBD [B.1.351]	86–112	78–123	80–126	90–125
SA-S [B.1.351]	91–120	83–127	87–113	90–125

Abbreviations: W-N, Wuhan Nucleocapsid; W-RBD, Wuhan receptor binding domain (RBD); W-S, Wuhan Spike (S); Br-RBD [P.1], Brazil RBD; Br-S [P.1], Brazil S; UK-RBD [B.1.1.7], United Kingdom RBD; UK-S [B.1.1.7], United Kingdom S; SA-RBD [B.1.351], South Africa RBD; SA-S [B.1.351], South Africa S.

**Table 7:** NIBSC reference panel (20/268) performance in MSD assay. Data are representative of NIBSC standard values expressed in BAU/mL using assay conversion factors. Values represent means, with the % CV values in parenthesis, as observed for six different runs.

	W-N		W-S		W-RBD	
	AU/mL	BAU/mL	AU/mL	BAU/mL	AU/mL	BAU/mL
**NIBSC High**	130,339 (14.1%)	308	94,318 (13.2%)	2047	21,859 (11.3%)	509
**NIBSC MID**	45,344 (12.9%)	107	27,350 (11.5%)	593	7971 (13.1%)	186
**NIBSC LOW**	4896 (10.8%)	12	3297 (9.9%)	72	790 (11.8%)	18
**Conversion factor**	0.00236		0.0217		0.0233	

Abbreviations: W-N, Wuhan Nucleocapsid; W-RBD, Wuhan receptor binding domain (RBD); W-S, Wuhan Spike.

**Table 8:** Serological signatures (Total IgG) in convalescent, breakthrough infected, and vaccinated non-infected groups. Median IgG concentrations in AU/mL observed among different groups. Comparisons were made among different groups using the Kruskal–Wallis test. *p* values of <0.05 were considered significant.

Sr. No.		W-S	Br-S [P.1]	UK-S [B.1.1.7]	SA-S [B.1.351]	W-RBD	Br-RBD [P.1]	UK-RBD [B.1.1.7]	SA-RBD [B.1.351]	W-N
Convalescent	Mean	153,142	96,724	149,779	107,637	61,700	44,362	57,755	41,650	121,550
	GM	38,340	24,558	40,571	25,313	9846	6166	9344	4221	60,727
	Min	585	368	521	385	244	146	243	143	1088
	Max	1,420,159	841,413	1,296,289	892,359	678,787	484,588	640,622	537,828	455,149
	Median	49,670	31,791	51,075	25,313	9846	6166	9344	3618	68,887
Breakthrough Infected	Mean	231,286	204,313	190,332	137,319	77,452	79,452	83,267	118,569	704
	GM	71,419	50,328	57,198	34,032	23,650	22,385	25,059	20,413	380
	Min	4515	1344	3651	1700	918	830	1047	618	47
	Max	1,170,950	1,031,308	976,125	680,803	459,708	483,341	518,697	874,411	2649
	Median	94,780	68,982	82,654	54,032	35,780	35,951	36,734	29,289	568
Vaccinated and Non-Infected	Mean	8555	4303	6347	4131	2366	2097	2610	1504	365
	GM	6707	3306	5056	3468	1813	1607	2020	1095	321
	Min	2433	976	2045	1577	512	634	596	287	160
	Max	15,193	10,680	14,085	7598	5426	5440	6248	3923	1040
	Median	7211	3438	5040	3517	1936	1495	2083	1117	361
*p*-value [One Way ANOVA]		*p* < 0.001	*p* < 0.001	*p* < 0.001	*p* < 0.01	*p* < 0.01	*p* < 0.01	*p* < 0.01	*p* < 0.01	*p* < 0.001

Abbreviations: W-N, Wuhan Nucleocapsid; W-RBD, Wuhan receptor binding domain (RBD); W-S, Wuhan Spike (S); Br-RBD [P.1], Brazil RBD; Br-S [P.1], Brazil S; UK-RBD [B.1.1.7], United Kingdom RBD; UK-S [B.1.1.7], United Kingdom S; SA-RBD [B.1.351], South Africa RBD; SA-S [B.1.351], South Africa S.

**Table 9:** Spike: Nucleocapsid IgG antibody ratios for developing serological signatures. Spike: Nucleocapsid IgG antibodies ratios were compared using the Kruskal–Wallis test. *p* values of <0.05 were considered significant.

	Spike: Nucleocapsid IgG Antibody Ratios							
	W-S: W-N	Br-S [P.1]: W-N	UK-S [B.1.1.7]: W-N	SA-S [B.1.351]: W-N	W-RBD: W-N	Br-RBD [P.1]: W-N	UK-RBD [B.1.1.7]: W-N	SA-RBD [B.1.351]: W-N
Convalescent	0.72	0.46	0.74	0.37	0.14	0.09	0.14	0.05
Breakthrough Infected	166.97	121.52	145.61	95.19	63.03	63.33	64.71	51.6
Vaccinated and Non-Infected	20	9.54	13.98	9.75	5.37	4.15	5.78	3.1
*p*-value [One Way ANOVA]	*p* < 0.001	*p* < 0.001	*p* < 0.001	*p* < 0.001	*p* < 0.001	*p* < 0.001	*p* < 0.001	NS

Abbreviations: W-N, Wuhan Nucleocapsid; W-RBD, Wuhan receptor binding domain (RBD); W-S, Wuhan Spike (S); Br-RBD [P.1], Brazil RBD; Br-S [P.1], Brazil S; UK-RBD [B.1.1.7], United Kingdom RBD; UK-S [B.1.1.7], United Kingdom S; SA-RBD [B.1.351], South Africa RBD; SA-S [B.1.351], South Africa S; NS, Non-significant.

**Table 10:** Serological signatures (functional antibodies) in convalescent, breakthrough-infected and vaccinated non-infected groups. Percentage inhibition was calculated relative to the assay calibrator (maximum 100% inhibition) using the equation below: % Inhibition = [1 − (Average Sample ECL Signal/Average ECL signal of Calibrator 8)] × 100; The percent inhibition levels for the anti-SARS-CoV-2 antibody were compared by using the Kruskal–Wallis test. *p* values of <0.05 were considered significant.

ACE-2 Neutralization	W-S	Br-S [P.1]	UK-S [B.1.1.7]	SA-S [B.1.351]	W-RBD	Br-RBD [P.1]	UK-RBD [B.1.1.7]	SA-RBD [B.1.351]	W-N
Convalescent	93.9	68.1	83.5	70.7	93.6	62.7	86.4	52.0	56.5
Breakthrough Infected	99.0	92.8	95.9	93.8	98.5	93.0	95.8	97.1	58.0
Vaccinated and Non-Infected	30.5	12.0	19.6	10.8	21.3	22.4	17.4	54.0	52.8
*p*-value [One Way ANOVA]	*p* < 0.001	*p* < 0.001	*p* < 0.01	*p* < 0.001	*p* < 0.001	NS	*p* < 0.05	*p* < 0.05	NS

Abbreviations: W-N, Wuhan Nucleocapsid; W-RBD, Wuhan receptor binding domain (RBD); W-S, Wuhan Spike (S); Br-RBD [P.1], Brazil RBD; Br-S [P.1], Brazil S; UK-RBD [B.1.1.7], United Kingdom RBD; UK-S [B.1.1.7], United Kingdom S; SA-RBD [B.1.351], South Africa RBD; SA-S [B.1.351], South Africa S; NS, Non-significant.

**Table 11:** Impact of the vaccine on different variants. Ratios in different groups were compared using the Kruskal–Wallis test. *p* values of <0.05 were considered to be significant.

	ACE-2 Neutralization			IgG		
	Br-S [P.1]: W-S	UK-S [B.1.1.7]: W-S	SA-S [B.1.351]: W-S	Br-S [P.1]: W-S	UK-S [B.1.1.7]: W-S	SA-S [B.1.351]: W-S
Convalescent	0.72	0.89	0.75	0.64	1.03	0.51
Breakthrough Infected	0.94	0.97	0.95	0.73	0.87	0.57
Vaccinated and Non-Infected	0.39	0.64	0.35	0.48	0.7	0.49
*p*-value [One Way ANOVA]	*p* < 0.01	*p* < 0.01	*p* < 0.001	*p* < 0.01	*p* < 0.01	*p* < 0.05

Abbreviations: W-S, Wuhan Spike (S); Br-S [P.1], Brazil S; UK-S [B.1.1.7], United Kingdom S; SA-S [B.1.351], South Africa S.

**Table 12:** Hematological parameters. Hematological parameters were compared among groups using Student’s t-test. *p* values of <0.05 were considered to be significant.

Parameter	Convalescent	Breakthrough Infected	*p*-Value [Student *t*-Test]
Ct Value	23	18	*p* < 0.01
Hemoglobin (g/dL)	12.02	14.9	*p* < 0.001
MCV (µm^3^)	90.21	88	*p* < 0.01
WBC (/mm^3^)	10,600	7200	*p* < 0.001
Neutrophils (%)	70	60	*p* < 0.001
Lymphocytes (%)	28	34	*p* < 0.001
Platelet (/µL)	242,000	286,000	NS
D-dimer (ng/mL)	739.34	119	*p* < 0.001
Ferritin(ng/mL)	370.33	149	*p* < 0.001
LDH (U/L)	717.99	379	*p* < 0.001
CRP (mg/L)	25	6.57	*p* < 0.01

## Data Availability

Data is contained within the article or Supplementary Materials.

## Supplementary Materials

The following supporting information can be downloaded at: https://www.mdpi.com/article/10.3390/vaccines12040433/s1, Supplementary Table S1. Antigen details used for specificity study; Supplementary Table S2. Values of Reference Standard 1 in MSD arbitrary units (AU/mL); Supplementary Table S3. Assigned values (AU/mL) for sera panel.
