# Supplementary material for: Validation and Suitability Assessment of Multiplex Mesoscale Discovery Immunogenicity Assay for Establishing Serological Signatures Using Vaccinated, Non-Vaccinated and Breakthrough SARS-CoV-2 Infected Cases
Source: Vaccines (Basel). 2024 Apr 18;12(4):433. doi: 10.3390/vaccines12040433 (PMC11053742; doi:10.3390/vaccines12040433)
Supplement: Supplementary file 1 [file vaccines-12-00433-s001.zip › vaccines-2826329-supplementary.pdf]

Supplementary Table S1
Antigen details used for specificity study

Antigen details	Batch. no.	Source	Storage(°C)
**Wuhan variant virus (Working stock)**	14141D001	In-House	-196 or below
**CRM197 protein**	IHWS/0.4mg/ml CRM/20/01	In-House	-20

Blank human serum details

Details	Batch. no.	Product No.	Source	Storage
**Blank human serum**	0000083106	S5393	Sigma	2-8° C.

Supplementary Table S2: Values of Reference Standard 1 in MSD Arbitrary units (AU/ml).

Antigens	CAL-01	CAL-02	CAL-03	CAL-04	CAL-05	CAL-06	CAL-07	CAL-08
**W-S**	72	18	4.5	1.13	0.281	0.0703	0.0176	0
**SA-RBD (B.1.351)**	4.4	1.1	0.275	0.0688	0.0172	0.0043	0.00107	0
**W-N**	70	17.5	4.38	1.09	0.273	0.0684	0.0171	0
**Br-RBD (P.1)**	7.0	1.75	0.438	0.109	0.0273	0.00684	0.00171	0
**UK-RBD (B.1.1.7)**	18	4.5	1.13	0.281	0.0703	0.0176	0.00439	0
**Br-S (P.1)**	29	7.25	1.81	0.453	0.113	0.0283	0.00708	0
**UK-S (B.1.1.7)**	44	11	2.75	0.688	0.172	0.043	0.0107	0
**SA-S (B.1.351)**	25	6.25	1.56	0.391	0.0977	0.0244	0.0061	0
**W-RBD**	29	7.25	1.81	0.453	0.113	0.0283	0.00708	0

W-N, Wuhan Nucleocapsid; W-RBD, Wuhan receptor binding domain (RBD); W-S, Wuhan Spike (S); Br-RBD [P.1], Brazil RBD; Br-S [P.1], Brazil S; UK-RBD [B.1.1.7], United Kingdom RBD; UK-S [B.1.1.7], United Kingdom S; SA-RBD [B.1.351], South Africa RBD; SA-S [B.1.351] South Africa S.

Supplementary Table S3: Assigned values (AU/ml) for sera panel

Sample ID	W-N	W-S	W-RBD	UK-S [B.1.1.7]	UK-RBD [B.1.1.7]	SA-S [B.1.351]	SA-RBD [B.1.351]	Br-S [P.1]	Br-RBD [P.1]
**Sample 1**	7962	47192	15314	42996	15076	25790	3978	26232	6513
**Sample 2**	206	2740	878	1794	953	1474	637	1562	737
**Sample 3**	11168	35902	10717	34240	8799	10131	4247	19532	7065
**Sample 4**	974	2960	1207	2267	1257	2125	801	1970	1144
**Sample 5**	93756	493127	236403	482255	263854	351300	174488	357748	250290
**Sample 6**	40675	52522	21619	48303	21832	50230	14690	34269	17854
**Sample 7**	4896	3297	790	2334	694	1882	233	2961	352
**Sample 8**	45344	27350	7971	17918	5658	9616	2155	11526	3381
**Sample 9**	130339	94318	21859	79280	20147	61017	5195	58047	9520

W-N, Wuhan Nucleocapsid; W-RBD, Wuhan receptor binding domain (RBD); W-S, Wuhan Spike (S); Br-RBD [P.1], Brazil RBD; Br-S [P.1], Brazil S; UK-RBD [B.1.1.7], United Kingdom RBD; UK-S [B.1.1.7], United Kingdom S; SA-RBD [B.1.351], South Africa RBD; SA-S [B.1.351] South Africa S.
